# Cross-cultural care awareness and self-perceived skillfulness among medical students in Egypt: a national study

**DOI:** 10.1186/s12909-026-10494-2

**Published:** 2026-10-31

**Authors:** Tasneem Deibes, Adham Hegazy, Abdallah Adel Nofal, Ahmed Abdelmageed, Shahd Aliwaisi, Mayar F. Elmenyawi, Sara Abdelaziz, Mahmoud W. Elaraby, Yousef Wael, Mustafa M. Badr, Mostafa R. Kamal, Jihan Magdi, Abdulrahman M. Elrefaey, ECCC Consortium, Sara Mohamed Roshdy Abdelwahed, Salma Moustafa Ebrahim Bashar, Marwan Mohammed Hegazy, Rania Mohammed Elshafiy, Nesma Khaled Abdelbadiaa Raid, Amany Ebrahim Fahmy Ebrahim, Zahraa Eissa Mohamed, Tasnim Omar Ghanoum, Hussam Saad Hamdi, Veronia Tawadrous, Sohaila A. Ibrahim, Judy Abo El Seoud, Mazen Essam, Saad Aboul Fetouh Saad Shalaby, Ahmed Awad Algamal, Shahd S. Hendawy, Obaida Hussein Mohammed, Youssef Khaled Sobhy Ahmed, Ahmed Mohamed Refaat Mesalm Alazb, Merna Ebrahim Abdelwahab, Alyaa S. Moselhy

**Affiliations:** 1 Faculty of Medicine, Mansoura University, Mansoura, Egypt; 2 Mansoura Manchester Program for Medical Education, Faculty of Medicine, Mansoura University, Mansoura, Egypt; 3 Faculty of Medicine, Mansoura Students’ Scientific Association, Mansoura University, Mansoura, Egypt; 4 Public Health and Community Medicine Department, Faculty of Medicine, Mansoura University, Mansoura, Egypt

**Keywords:** Cultural competence, Cultural awareness, Cultural humility, Health disparities, Medical education

## Abstract

**Background:**

Cross-cultural care (CCC) enables effective, patient-centered care for individuals from diverse backgrounds. Despite its importance in improving care quality and reducing health disparities, evidence on CCC among medical students in Egypt remains scarce. This study assessed medical students' cross-cultural self-perceived skillfulness and cultural awareness, examined their association, and identified sociodemographic and educational factors linked to both.

**Methods:**

A multicentric cross-sectional study was conducted between November 2025 and March 2026 across 14 Egyptian medical schools. Using convenience sampling, 1,645 medical students from all academic years and interns participated. Data were collected using an online questionnaire that included sociodemographic and educational factors, the Modified Cultural Awareness Scale (mCAS), and the Harvard CCC Survey skillfulness subscale. Statistical analyses included correlation, group comparisons, and multivariable linear regression.

**Results:**

Among 1,645 participants (mean age 21.8 ± 1.9 years; 53.7% female), the mean self-perceived cross-cultural skillfulness score was 3.55/5 (SD 0.78). Students felt most skilled in identifying a patient's Arabic literacy and taking a social history, and least skilled in counseling on complementary and alternative medicine and identifying patient mistrust. The mean total cultural awareness score was 4.69/7 (SD 0.75), highest in the Cognitive Awareness and Clinical Issues domains and lowest in General Educational and Research Experience. Higher awareness was associated with advanced English fluency and greater perceived adequacy of clinical experience and training (all *p* < 0.001), and skillfulness correlated positively with total awareness (*r* = 0.383, *p* < 0.001). In multivariable analysis, advanced English fluency, prior cultural sensitivity training, and all four cultural awareness domains were independently associated with skillfulness (all *p* ≤ 0.036).

**Conclusion:**

Among medical students in Egypt, self-perceived cross-cultural skillfulness was at an intermediate level and was independently associated with advanced English fluency, prior cultural sensitivity training, and greater cultural awareness across all mCAS domains. These findings identify cultural sensitivity training and cultural exposure as actionable targets for strengthening cross-cultural skillfulness in the medical curriculum.

**Supplementary Information:**

The online version contains supplementary material available at https://doi.org/10.1186/s12909-026-10494-2.

## Introduction

Cross-cultural care (CCC) refers to the ability of healthcare providers to deliver effective, individualized care to patients from diverse cultural, religious, linguistic, and social backgrounds while respecting their beliefs, values, and identities [1]. Growing cultural diversity and persistent healthcare disparities have highlighted the importance of preparing physicians to provide care that is responsive to patients' cultural contexts [2, 3]. Two key components underpin effective CCC: cultural awareness and cultural skillfulness. Cultural awareness refers to healthcare providers' recognition of their own cultural perspectives, assumptions, and biases, as well as their understanding of how cultural differences influence patients' health beliefs and behaviors [4]. Cultural skillfulness represents the practical ability to incorporate this understanding into clinical practice, such as eliciting culturally relevant information, communicating effectively across cultural differences, and adapting management plans to patients' cultural needs [5]. Underpinning both is cultural humility, a lifelong orientation of self-reflection and openness that values ongoing learning over a sense of mastery [6, 7]. Together, cultural awareness and skillfulness contribute to the delivery of equitable and culturally responsive healthcare [4].

CCC is widely promoted as a strategy to improve the quality of care delivered to culturally and linguistically diverse populations and to reduce racial and ethnic health disparities [3, 8]. When these capacities are lacking, the consequences are equally significant; when providers are insensitive to cultural or linguistic differences, diverse patients access services less readily, encounter communication barriers, and experience lower-quality care and poorer health outcomes [3, 9]. Crucially, evidence points towards significant gaps in both the teaching and acquisition of these competencies during undergraduate medical training. Medical students across diverse settings consistently self-report deficiencies in cross-cultural skills that persist through their final year, with inadequate cross-cultural education repeatedly identified as the primary barrier [5, 10].

In practice, despite CCC becoming an expected element of medical curricula globally, how it is taught varies widely, from longitudinal integration across all years to a single early block delivered without reinforcement and, frequently, without any national standard [5, 11]. This inconsistency is evident in students’ preparedness. For instance, fewer than half of fourth-year students at Harvard Medical School rated themselves skilled on most cultural skillfulness tasks, meanwhile, in Ireland, roughly half felt unprepared to care for ethnic-minority patients [11]. While in Nepal, most students reported favorable attitudes toward cultural diversity, yet themselves called for multicultural content to be formally incorporated into the curriculum [12]. These shared gaps recurring across diverse settings point to shared curricular defects rather than isolated local deficiencies [5, 10].

Egypt represents a distinctive yet understudied setting for CCC. Its patient population is culturally heterogeneous, marked by wide variation in religiosity, health literacy [13], and rural–urban divides [14], alongside a strong reliance on complementary and alternative medicine (CAM) [15]. Additionally, Egyptian universities have become regional educational hubs, hosting roughly 51,600 international students in 2024, many of whom experience cultural isolation and difficulty adapting to their new environment, while domestic medical students themselves report challenges in multicultural learning settings [16, 17]. Against this backdrop, Egypt has recently reformed its undergraduate medical curriculum toward a competency-based, integrated model intended to graduate physicians prepared to care for a diverse patient population, making it relevant to examine how well students are developing cross-cultural awareness and skillfulness [18]. The national evidence is also scarce, with a single institution study reporting generally high intercultural sensitivity among third-year students; yet, higher sensitivity was significantly associated with being an international student, greater English proficiency, and prior intercultural exposure, implying that domestic students may require more targeted support [17]. More broadly, cultural competence research in the Arab region remains limited, particularly regarding care for the region's specific minority and cross-border patient populations, including Egypt's Nubian, Bedouin, and Coptic communities, alongside its substantial Sudanese and Syrian patient populations, and largely relies on evidence from high-income Western settings [19, 20].

Given the limited research on CCC among medical students in Egypt and the wider Arab region, this study assessed Egyptian medical students’ self-perceived cultural skillfulness and awareness, examined the relationship between skillfulness and cultural awareness domains, and identified sociodemographic and educational factors associated with these outcomes. By addressing this gap, the study aims to offer insights from an underrepresented context to inform future curricular reform efforts.

## Methods

### Study design

This was a multicentric, descriptive cross-sectional study with an analytical component, conducted across medical schools in Egypt between November 2025 and March 2026. The study was designed and reported in accordance with the Strengthening the Reporting of Observational Studies in Epidemiology (STROBE) [21] guidelines which is attached as supplementary material (S1 File). Ethical approval for this study was granted by the Institutional Review Board of the Faculty of Medicine, Mansoura University with the approval code R.25.08.3298, and all procedures were conducted in accordance with the principles of the Declaration of Helsinki.

### Population and setting

The undergraduate medical program in Egypt follows a 5 + 2 structure: five academic years followed by a compulsory two-year clinical internship required for graduation. Following the 2018 medical education reform [18], the academic program was redesigned as an integrated curriculum in which institutions are encouraged to offer early clinical experience (ECE) during the preclinical years, with clinical rotations across the later years culminating in the mandatory two-year internship. To explore the effect of clinical exposure on CCC, this study included undergraduate medical students at all stages of training, from the first through fifth academic years, as well as interns.

Egypt's medical education system is administered across three higher education sectors, collectively comprising 50 medical schools at the time of this study's conception. These sectors differ in governance, curricula, and patient population served: government-run public universities (24 schools; 70.1% of students), private universities (23 schools; 23.4% of students), and Al-Azhar University; an Islamic institution operating under a distinct curriculum framework with separate male and female faculties (3 schools; 6.5% of students) [22]. To enhance representativeness across sectors and enable comparisons of cultural awareness and self-perceived skillfulness by institutional background, participants were recruited from 14 universities spanning all three sectors; eight universities from the public sector, four universities from the private sector and two out of the three Azhar University branches.

To capture geographic differences and to reduce sampling bias, we recruited from a geographically diverse set of universities representing different regions and socioeconomic backgrounds. The 14 universities spanned Egypt's four main regions: Upper Egypt (Minya and Sohag Universities), Urban governorates (Cairo, Alexandria, Galala, Misr University for Science and Technology [MUST], and Al-Azhar–Cairo Universities), Lower Egypt (Mansoura, Kafr El-Sheikh, Tanta, Delta, and Al-Azhar–Damietta Universities), and Frontier governorates (New Valley and King Salman International Universities). To maintain representativeness, the total required sample was allocated proportionately across these universities based on their official student census [22]. The selection of participating institutions was guided by the availability of local collaborators for data collection.

Inclusion criteria were: current enrollment at one of the 14 participating Egyptian medical schools, active status in any academic year from first year through the two internship years, and provision of informed consent. Students who did not provide informed consent did not proceed to the survey and were therefore not enrolled as participants. Among those who did consent, entries were excluded if the respondent was not enrolled at one of the participating institutions at the time of data collection, had already completed their internship and graduated, or had provided incomplete responses. On this basis, 57 entries were excluded from the preliminary dataset of 1702 responses, yielding a final dataset of 1645.

### Sample size

The sample size was calculated in RStudio using the sampling book package [23]. Using a Standard Deviation (SD) of 0.614 for the mean overall self-perceived skillfulness score based on the results of a preliminary pilot study on 30 medical students. Assuming a 95% confidence level and a 0.05 margin of error, the required sample size was calculated as 578. To account for clustering by university, a design effect of 2.5 was applied which is within the range of 2 to 6 commonly reported for multi-site surveys in developing-country settings [24], increasing the required minimum sample size to 1,445. A total of 1,645 responses were ultimately included in the analysis.

### Sampling and data collection approach

This study employed a proportional convenience sampling technique, given the practical constraints and the absence of a feasible method to contact all medical students for simple random sampling. Data were collected using a self-administered online questionnaire on Google Forms. The form link was distributed through official institutional channels and groups on Telegram and other social media platforms of the included Egyptian medical schools, with collaborators across Egypt recruited for data collection. At least one collaborator from each included medical school was responsible for overseeing the data collection process, disseminating the questionnaire, and making it accessible to students of all academic years. The form was also set to accept one response per Google account, hence preventing duplicate entries. Because the questionnaire was distributed through open institutional and social media channels without a fixed denominator of students reached, a precise response rate could not be calculated. Prior to widespread distribution, a pilot study was conducted with 30 medical students to evaluate the clarity and suitability of the questionnaire items and to determine the standard deviation (SD) of the skillfulness scale for sample size calculation. Based on the feedback received, minor revisions were made to the final questionnaire, and all pilot data were excluded from the final analysis.

### Study tools

Data were collected using a structured, self-reported online questionnaire to assess cultural awareness, self-perceived cultural skillfulness, and their influencing factors. The questionnaire was administered in English, as it is the primary language of instruction across Egyptian medical schools and to ensure accessibility for the non-Egyptian students in the sample, who may not be proficient in written Arabic. The questionnaire consisted of three primary sections. The first section gathered informed consent of participation in the study, along with sociodemographic and academic information, including age, sex, academic year, university, nationality, place of residence, self-reported English language proficiency, prior exposure to cultural sensitivity training, extracurricular activity involvement, previous participation in medical training abroad, and perceived adequacy of both ECE and clinical training.

The second section evaluated participants' cultural awareness utilizing the Modified Cultural Awareness Scale (mCAS) [4]. The scale comprises 35 items structured on a 7-point Likert scale, with responses ranging from “1 = strongly disagree” to “7 = strongly agree”. The mCAS has four distinct subscales each measuring a specific domain of cultural awareness: General Educational and Research Experience, Cognitive Awareness, Clinical Issues, and Behaviors/Comfort with Interactions. Domain scores were then computed as the mean of their constituent items, and the total mCAS score was calculated as the mean of all 35 items, yielding a possible range of 1 to 7 for both domain and total scores, with higher scores indicating greater cultural awareness. Prior to scoring, negatively worded items were reverse-coded so that higher scores consistently reflect greater cultural awareness across all domains. The mCAS has previously demonstrated robust internal consistency, yielding a total scale Cronbach alpha of 0.88 and subscale alphas ranging from 0.70 to 0.90 among undergraduate health and social care students. In our sample, internal consistency was similarly high, with a total Cronbach alpha of 0.92 and subscale alphas ranging from 0.77 to 0.89 [25]. These values indicate that items within each domain correlate well with one another in the present sample but do not, by themselves, establish the validity of the modified instrument in the Egyptian context.

The third section measured self-perceived clinical skillfulness in delivering CCC using the skillfulness subscale of the Harvard Cross-Cultural Care Survey (CCCS) [5]. This subscale asks participants to rate their skill level across ten distinct clinical scenarios on a 5-point Likert scale, ranging from “not at all skillful” to “very skillful”. Higher scores indicate a greater perceived ability to navigate cross-cultural clinical interactions, such as working effectively with medical interpreters, assessing patient understanding, and negotiating treatment plans. Internal consistency for skillfulness subscale was also confirmed by a Cronbach of 0.894 in our sample.

Following a pilot testing phase, minor modifications were introduced to enhance the cultural and contextual appropriateness of the instruments for an Egyptian medical student cohort. To ensure clarity, Arabic translations and explanatory clarifications were added as a question description to two specific items within the mCAS. These included the fifth item of the cognitive awareness domain, assessing whether instructors or supervisors call on students from minority cultural groups when relevant issues arise, and the seventh item of the clinical issues domain, evaluating whether instructors or supervisors model behaviors sensitive to multicultural issues. Furthermore, to better reflect the linguistic demographic of the study population as an Arabic native country, the tenth item in the skillfulness subscale assessing the ability to identify how well a patient can read or write English was adapted to specify “Arabic” instead of “English”. The appropriateness of these modifications was reviewed by a medical education researcher prior to survey launch, consistent with the emphasis on expert committee review in established guidance on the cross-cultural adaptation of self-report measures [26]. To further assess whether these modifications affected the psychometric behavior of their respective items, corrected item-total correlations were calculated for each of the three modified items relative to their parent subscale, along with subscale Cronbach's alpha if each item were removed. All three modified items showed corrected item-total correlations exceeding the conventional 0.30 threshold for a meaningful item-scale contribution [27]: 0.45 for the cognitive awareness item, 0.38 for the clinical issues item, and 0.57 for the substituted skillfulness item; and removal of any single item did not meaningfully alter subscale-level Cronbach's alpha (0.83 → 0.82, 0.81 → 0.81, and 0.89 → 0.89, respectively), indicating that the modifications did not detach these items from their underlying constructs.

### Statistical analysis

Statistical analysis was conducted using Jamovi statistical software [28]. Descriptive statistics were used to summarize participant characteristics and academic data; continuous variables were summarized as means and SDs as the data was found to be normally distributed on visual inspection of quantile–quantile (Q-Q) plots, while categorical variables were reported as frequencies and percentages. For the clinical skillfulness items, individual 5-point Likert scale responses were summarized both continuously as mean scores and categorically by calculating the frequencies and percentages of respondents achieving 'Skilled' (score of 4 or 5) and 'Intermediate' (score of 3) status, consistent with the reporting convention used in the original development and validation of the Harvard Cross-Cultural Care Survey skillfulness subscale [5].

For inferential statistics, Pearson correlation coefficients (r) were calculated to examine the bivariate relationships between the mCAS domain scores, the total mCAS score, self-perceived skillfulness scores, and participants' age. Comparisons of mean scores across various sociodemographic characteristics were performed using independent samples t-tests for two-group comparisons and one-way ANOVA for multi-group comparisons. Effect sizes were calculated for all statistically significant comparisons, using Cohen's d for two-group comparisons and eta-squared (η^2^) for comparisons involving more than two groups, and are reported alongside the corresponding p-values.

Factors independently associated with self-perceived skillfulness scores were identified using a two-stage linear regression modeling approach. First, univariate linear regression models were run separately for each potential variable to assess its unadjusted association with skillfulness. Subsequently, a multivariable linear regression model was constructed including all measured variables simultaneously. All measured variables were entered into the multivariable model simultaneously, regardless of univariate significance, to avoid excluding genuine confounders whose association with the outcome only emerges once other variables are accounted for [29]. Categorical variables were entered into the model as dummy variables using the specified reference categories. Variance inflation factors (VIFs) were calculated for all variables included in the multivariable model to assess multicollinearity, with particular attention to the four mCAS domains given their simultaneous inclusion from a single instrument. Results were reported as unstandardized regression coefficients (B) with their corresponding 95% confidence intervals (CI). The overall explanatory power of the multivariable model was assessed using the coefficient of determination (R^2^). A *p*-value of < 0.05 was considered statistically significant for all analyses. A Harman's single-factor test was also conducted to assess the risk of common-method bias, given that the mCAS and skillfulness subscale were both self-reported by the same respondents in a single administration.

## Results

### Participants’ characteristics

A total of 1,645 medical students were included, with a mean age of 21.8 ± 1.88 years. The sample was predominantly female (884, 53.7%), Egyptian (1,471, 89.4%), and had an urban place of residence (1,128, 68.6%). The majority were enrolled at public universities (1,252, 76.1%) and affiliated with universities in the Urban Governorates (734, 44.6%) or Lower Egypt (664, 40.4%) regions (Table 1).

**Table 1:** Sociodemographic characteristics of the study participants (*N* = 1,645)

Characteristic	n (%)^1^
Age	21.8 (1.88)
Sex	
Male	761 (46.3%)
Female	884 (53.7%)
Academic Year	
1 st Year	94 (5.7%)
2nd Year	144 (8.8%)
3rd Year	288 (17.5%)
4th Year	234 (14.2%)
5th Year	466 (28.3%)
Interns	419 (25.5%)
University Type	
Public	1252 (76.1%)
Private	124 (7.5%)
Al-Azhar	269 (16.4%)
Region of University	
Lower Egypt	664 (40.4%)
Urban	734 (44.6%)
Upper Egypt	197 (12.0%)
Frontier Governorates	50 (3.0%)
Nationality	
Egyptian	1471 (89.4%)
Non-Egyptian	174 (10.6%)
Residence	
Rural	517 (31.4%)
Urban	1128 (68.6%)
English Fluency	
Basic/Conversational	271 (16.5%)
Intermediate/Working	818 (49.7%)
Advanced/Fluent	556 (33.8%)
Cultural Sensitivity Training	
Yes	569 (34.6%)
No	1076 (65.4%)
Extra-curricular Activities Involvement	
Yes	946 (57.5%)
No	699 (42.5%)
Previous Participation in Medical Training Abroad	
Yes	355 (21.6%)
No	1290 (78.4%)
Perceived Adequacy of Early Clinical Experience	
Less than Adequate	492 (29.9%)
Adequate	837 (50.9%)
More than Adequate	161 (9.8%)
Did not have ECE in my pre-clinical years	155 (9.4%)
Perceived Adequacy of Clinical Training	
Less than Adequate	413 (25.1%)
Adequate	832 (50.6%)
More than Adequate	196 (11.9%)
Not in my clinical years yet	204 (12.4%)

^1^Data are presented as mean (SD) for continuous variables and n (%) for categorical variables

Clinically advanced students, fifth-year students, and interns constituted the largest academic year groups (466, 28.3%, and 419, 25.5%, respectively). English proficiency was intermediate or working for nearly half the sample (818, 49.7%), with advanced/fluent proficiency in one-third (556, 33.8%). Notably, only 569 students (34.6%) reported having received prior cultural sensitivity training. More than half were involved in extracurricular activities (946, 57.5%), and 355 (21.6%) had participated in medical training abroad. Regarding clinical training perceptions, approximately half rated both their early clinical experience (837, 50.9%) and their overall clinical training (832, 50.6%) as adequate (Table 1).

### Cross-cultural care self-perceived skillfulness

The mean overall self-perceived skillfulness score was 3.55 ± 0.78. Item-level scores ranged from 3.42 to 3.70. The highest-scoring items were identifying a patient's Arabic literacy (3.70 ± 1.10), taking a social history (3.68 ± 1.08), and determining how a patient prefers to be addressed and interacted with (3.66 ± 1.02), with 58.5%, 58.9%, and 57.7% of participants receiving a Skilled rating, respectively (Table 2).

**Table 2:** Distribution of clinical skillfulness scores among Egyptian medical students as assessed by the Harvard cross-cultural care survey skillfulness sub-scale (*N* = 1,645)

How much are you skilled in:	% Skilled^1^	% Intermediate^2^	Mean Score (SD)^3^
Assessing the patient's understanding of the cause of his or her illness	834 (50.7)	537 (32.6)	3.48 (1.08)
Working effectively with a medical interpreter	832 (50.5)	523 (31.8)	3.48 (1.13)
Negotiating with the patient about key aspects of the treatment plan	922 (56)	495 (30.1)	3.60 (1.04)
Determining how a patient wants to be addressed and interacted with	947 (57.7)	496 (30.2)	3.66 (1.02)
Taking a social history	970 (58.9)	441 (26.8)	3.68 (1.08)
Identifying whether a patient is mistrustful of the healthcare system or physicians	809 (49.2)	556 (33.8)	3.46 (1.07)
Counseling patients about their use of complementary or alternative medicine	797 (48.5)	526 (32)	3.42 (1.10)
Identifying religious beliefs that might affect clinical care	877 (53.3)	455 (27.7)	3.53 (1.16)
Identifying cultural (nonreligious) customs that might affect clinical care	843 (51.3)	485 (29.5)	3.46 (1.12)
Identifying how well a patient can read or write Arabic	963 (58.5)	457 (27.8)	3.70 (1.10)

Items are scored on a 5-point Likert scale (1 = not skilled at all to 5 = extremely skilled). Frequencies for % Skilled and % Intermediate are expressed as n (%)

^1^Skilled; score of 4 or 5

^2^Intermediate; score of 3

^3^Mean Score (SD) reflects the mean item score across all participants

In contrast, the lowest-scoring items were counseling patients about their use of CAM (3.42 ± 1.10; 48.5% skilled), identifying whether a patient is mistrustful of the healthcare system or physicians (3.46 ± 1.07; 49.2% skilled), and identifying cultural (nonreligious) customs that may affect clinical care (3.46 ± 1.12; 51.3% skilled). Notably, fewer than half of participants reported being skilled in counseling about CAM use and recognizing patient mistrust, the only two items with skillfulness rates below 50% (Table 2).

### Correlations between mCAS domains, skillfulness, and age

There was a positive correlation between the total mCAS score and self-perceived skillfulness (*r* = 0.383, *p* < 0.001). At the domain level, clinical issues demonstrated the strongest positive correlation with skillfulness (*r* = 0.416, *p* < 0.001), followed by general educational and research experience (*r* = 0.374, *p* < 0.001) and cognitive awareness (*r* = 0.336, *p* < 0.001). On the other hand, Behaviors/Comfort with Interactions was the only domain to show a negative correlation with skillfulness (*r* = − 0.104, *p* < 0.001) (Table 3). A Harman's single-factor test across all mCAS and skillfulness items showed that the first unrotated factor explained 25.4% of total variance, below the 50% threshold conventionally used to flag common-method bias as a likely dominant explanation.

**Table 3:** Pearson correlation matrix of mCAS domain scores, total mCAS score, skillfulness, and age among Egyptian medical students (*N* = 1,645)

Variable	1	2	3	4	5	6
1. General educational and research experience	—					
2. Behaviors/comfort with interactions	−0.050*	—				
3. Cognitive awareness	0.413***	−0.050*	—			
4. Clinical issues	0.519***	0.090***	0.578***	—		
5. Total mCAS Score	0.850***	0.332***	0.646***	0.734***	—	
6. Skillfulness	0.374***	−0.104***	0.336***	0.416***	0.383***	—
7. Age	−0.050*	0.012	−0.015	−0.045	−0.042	0.056*

*mCAS* Modified Cultural Awareness Scale

^*^p <.05

^***^p <.001

### Sociodemographic variations in cultural awareness and self-perceived skillfulness

The mean total mCAS and self-perceived skillfulness scores were 4.69 ± 0.75 and 3.55 ± 0.78, respectively. Among the mCAS domains, Cognitive Awareness and Clinical Issues had the highest mean scores (both 4.92), whereas General Educational and Research Experience had the lowest (4.48). Sex was not associated with total mCAS or skillfulness scores, although females scored slightly higher on Behaviors/Comfort with Interactions (*p* = 0.035). The academic year was also associated with Behaviors/Comfort with interactions (*p* = 0.048) and skillfulness (*p* = 0.030, negligible effect size), with interns reporting the highest and first-year students the lowest skillfulness scores. Higher English proficiency was associated with greater cultural awareness and skillfulness (both *p* < 0.05,) (Table 4).

**Table 4:** Comparison of mCAS domain scores, total mCAS score, and skillfulness scores across sociodemographic characteristics among Egyptian medical students (*N* = 1,645)

Variable	Mean Score (SD)					
	General educational and research experience	Behaviors/comfort with interactions	Cognitive awareness	Clinical issues	Total mCAS Score	Skillfulness mean
Overall	4.48 (1.14)	4.75 (1.19)	4.92 (1.05)	4.92 (1.09)	4.69 (0.75)	3.55 (0.78)
Sex						
Male	4.48 (1.13)	4.68 (1.22)	4.91 (1.01)	4.90 (1.12)	4.67 (0.75)	3.57 (0.78)
Female	4.49 (1.14)	4.81 (1.16)	4.93 (1.02)	4.93 (1.06)	4.71 (0.76)	3.53 (0.78)
* p*-value (effect size)	0.881	**0.035 (0.105)**	0.744	0.591	0.289	0.282
Academic Year						
1 st Year	4.72 (1.20)	4.64 (1.33)	4.89 (1.08)	4.95 (1.17)	4.77 (0.84)	3.43 (0.87)
2nd Year	4.58 (1.32)	4.53 (1.34)^a^	4.84 (1.17)	4.89 (1.20)	4.67 (0.90)	3.54 (0.86)
3rd Year	4.48 (1.13)	4.73 (1.20)	5.02 (1.04)	4.93 (1.13)	4.71 (0.721)	3.50 (0.80)
4th Year	4.53 (1.19)	4.83 (1.29)	4.91 (1.10)	5.04 (1.06)	4.75 (0.74)	3.57 (0.81)
5th Year	4.43 (1.11)	4.70 (1.10)	4.84 (1.07)	4.81 (1.07)	4.63 (0.77)	3.50 (0.73)^a^
Interns	4.43 (1.06)	4.87 (1.12)^a^	4.98 (0.05)	4.95 (1.02)	4.72 (0.69)	3.65 (0.75)^a^
*p*-value (effect size)	0.237	**0.048 (0.007)**	0.173	0.133	0.323	**0.03 (0.007)**
University Type						
Public	4.46 (1.13)	4.83 (1.19)^a^	4.93 (1.03)	4.90 (1.08)	4.70 (0.75)	3.55 (0.78)
Private	4.73 (1.30)	4.57 (1.29)	4.98 (1.05)	5.05 (1.14)	4.79 (0.80)	3.66 (0.82)
Al-Azhar	4.49 (1.08)	4.46 (1.09)^a^	4.86 (1.14)	4.93 (1.10)	4.62 (0.75)	3.48 (0.75)
*p*-value (effect size)	0.091	** <.001 (0.015)**	0.526	0.342	0.102	0.12
Region of University						
Lower Egypt	4.56 (1.13)	4.71 (1.22)	4.91 (1.08)	4.95 (1.13)	4.72 (0.78)	3.58 (0.74)
Urban	4.44 (1.11)	4.83 (1.15)^a^	4.97 (0.97)	4.92 (1.02)	4.70 (0.71)	3.53 (0.80)
Upper Egypt	4.34 (1.18)	4.67 (1.15)	4.83 (1.15)	4.82 (1.11)	4.58 (0.77)	3.51 (0.78)
Frontier Governorates	4.64 (1.35)	4.37 (1.30)^a^	4.77 (1.24)	4.80 (1.30)	4.63 (0.96)	3.51 (0.92)
*p*-value (effect size)	**0.047 (0.005)**	**0.026 (0.006)**	0.318	0.482	0.136	0.592
Nationality						
Egyptian	4.49 (1.14)	4.76 (1.19)	4.93 (1.06)	4.92 (1.09)	4.70 (0.76)	3.55 (0.78)
Non-Egyptian	4.45 (1.12)	4.63 (1.15)	4.82 (0.98)	4.87 (1.04)	4.63 (0.74)	3.56 (0.79)
*p*-value (effect size)	0.685	0.177	0.159	0.571	0.209	0.849
Residence						
Urban	4.45 (1.11)	4.83 (1.17)	4.98 (0.96)	4.93 (1.00)	4.71 (0.71)	3.55 (0.78)
Rural	4.57 (1.19)	4.58 (1.22)	4.81 (1.21)	4.87 (1.25)	4.66 (0.84)	3.54 (0.78)
*p*-value (effect size)	**0.041 (0.109)**	** <.001 (0.211)**	**0.002 (0.161)**	0.29	0.244	0.723
English Fluency						
Basic/Conversational	4.36 (1.28)^a^	4.35 (1.09)^a,b^	4.63 (1.22)^a,b^	4.53 (1.30)^a,b^	4.43 (0.85)^a,b^	3.42 (0.86)^a^
Intermediate/Working	4.56 (1.07)^a^	4.68 (1.18)^a,c^	4.94 (1.02)^a^	4.93 (1.02)^a,c^	4.71 (0.72)^a^	3.53 (0.73)
Advanced/Fluent	4.43 (1.15)	5.05 (1.17)^b,c^	5.04 (0.98)^b^	5.08 (1.02)^b,c^	4.79 (0.73)^b^	3.63 (0.80)^a^
*p*-value (effect size)	**0.021 (0.005)**	** <.001 (0.043)**	** <.001 (0.017)**	** <.001 (0.029)**	** <.001 (0.025)**	**0.003 (0.008)**
Cultural Sensitivity Training						
Yes	4.58 (1.23)	4.43 (1.25)	4.84 (1.18)	4.93 (1.22)	4.65 (0.81)	3.65 (0.76)
No	4.44 (1.08)	4.92 (1.12)	4.96 (0.97)	4.91 (1.01)	4.72 (0.72)	3.49 (0.78)
*p*-value (effect size)	**0.017 (0.124)**	** <.001 (0.413)**	**0.024 (0.117)**	0.683	0.069	** <.001 (0.204)**
Extra-curricular Activities Involvement						
Yes	4.49 (1.16)	4.71 (1.23)	4.95 (1.06)	4.97 (1.10)	4.70 (0.76)	3.60 (0.79)
No	4.48 (1.10)	4.80 (1.12)	4.89 (1.04)	4.84 (1.06)	4.69 (0.75)	3.47 (0.77)
*p*-value (effect size)	0.929	0.108	0.267	**0.01 (0.129)**	0.752	**0.001 (0.163)**
Previous Participation in Medical Training Abroad						
Yes	4.60 (1.29)	4.16 (1.27)	4.82 (1.28)	4.91 (1.32)	4.59 (0.84)	3.66 (0.87)
No	4.45 (1.09)	4.91 (1.11)	4.95 (0.98)	4.92 (1.01)	4.72 (0.73)	3.52 (0.75)
*p*-value (effect size)	**0.037 (0.125)**	** <.001 (0.658)**	**0.043 (0.121)**	0.888	**0.002 (0.184)**	**0.002 (0.188)**
Perceived Adequacy of Early Clinical Experience						
Less than Adequate	4.16 (1.00)^a,b^	4.92 (1.07)^a,b^	4.99 (1.01)	4.86 (0.99)	4.60 (0.68)^a^	3.46 (0.78)^a,b^
Adequate	4.68 (1.09)^a,c^	4.68 (1.19)^a,c^	4.89 (1.01)	4.95 (1.05)	4.76 (0.75)^a^	3.59 (0.72)^a,c^
More than Adequate	4.78 (1.42)^b,d^	4.40 (1.42)^b,d^	4.77 (1.36)	4.85 (1.57)	4.70 (0.98)	3.70 (0.91)^b,d^
Did not have ECE in my pre-clinical years	4.12 (1.17)^c,d^	4.95 (1.14)^c,d^	5.03 (1.04)	4.97 (0.99)	4.61 (0.70)	3.39 (0.88)^c,d^
*p*-value (effect size)	** <.001 (0.056)**	** <.001 (0.019)**	0.107	0.293	** <.001 (0.010)**	** <.001 (0.013)**
Perceived Adequacy of Clinical Training						
Less than Adequate	4.11 (1.03)^a,b,c^	4.77 (1.10)	4.95 (1.05)	4.84 (1.04)	4.53 (0.68)^a,b,c^	3.46 (0.76)^a,b^
Adequate	4.58 (1.10)^a^	4.72 (1.19)^a^	4.86 (1.01)	4.91 (1.06)	4.72 (0.75)^a^	3.58 (0.75)^a,c,d^
More than Adequate	4.80 (1.40)^b^	4.59 (1.41)^b^	4.98 (1.28)	4.99 (1.36)	4.82 (.093)^b^	3.76 (0.82)^b,c,e^
Not in my clinical years yet	4.53 (1.05)^c^	5.00 (1.11)^a,b^	5.05 (0.96)	5.01 (0.96)	4.82 (0.70)^c^	3.36 (0.83)^d,e^
*p*-value (effect size)	** <.001 (0.040)**	**0.005 (0.008)**	0.081	0.197	** <.001 (0.018)**	** <.001 (0.020)**

Data are presented as mean (SD). Independent samples t-test was used for two-group comparisons; one-way ANOVA was used for multi-group comparisons. Post hoc pairwise comparisons were conducted using Tukey's test for groups with significant ANOVA results

Shared superscript letters (a, b, c, d, e) indicate a statistically significant pairwise difference between those two groups (*p* < 0.05)

mCAS = Modified Cultural Awareness Scale

*p*-values < 0.05 are considered statistically significant and are flagged as bold

Effect sizes are reported next to the *p*-value for each statistically significant comparison: Cohen's d for two-group comparisons, η^2^ for comparisons involving more than two groups

Importantly, several experiential and training-related factors were associated with higher scores. Students who had received cultural sensitivity training reported higher skillfulness scores than those without such training (3.65 vs. 3.49, *p* < 0.001), while those involved in extracurricular activities also scored higher (3.60 vs. 3.47, *p* = 0.001). Perceived adequacy of both early clinical experience and overall clinical training was positively associated with total mCAS and skillfulness scores (all *p* < 0.001). Notably, cultural sensitivity training and prior medical training abroad were associated with lower behaviors/comfort with interactions scores (4.43 vs. 4.92 and 4.16 vs. 4.91, respectively; both *p* < 0.001) with training abroad showing the largest effect size among all sociodemographic comparisons (Table 4).

### Factors associated with cross-cultural care self-perceived skillfulness

Several factors were associated with self-perceived skillfulness in univariate analyses, while after adjustment in multivariable analysis, advanced English fluency (B = 0.118, *p* = 0.036), cultural sensitivity training (B = 0.086, *p* = 0.033), and not yet having begun clinical training (among the perceived clinical-training-adequacy categories) (B = − 0.155, *p* = 0.020) were identified as factors independently associated with skillfulness. All four mCAS domains were independently associated with skillfulness, with Clinical Issues showing the strongest positive association (B = 0.189, *p* < 0.001) and Behaviors/Comfort with Interactions the only negative association (B = − 0.067, *p* < 0.001). VIFs across all variables in the multivariable model ranged from 1.07 to 1.98, including 1.20 to 1.87 for the four mCAS domains specifically, indicating no problematic multicollinearity. The model explained 25.7% of the variance in self-perceived skillfulness scores (Table 5).

**Table 5:** Univariate and multivariable linear regression analysis of factors associated with skillfulness scores among Egyptian medical students (*N* = 1,645)

Variable	Univariate		Multivariable	
	B (95% CI)	p-value	B (95% CI)	*p*-value
Age	0.023 (0.003, 0.043)	**0.023**	0.012 (−0.023, 0.047)	0.492
Sex				
Male	ref		ref	
Female	−0.042 (−0.117, 0.034)	0.282	−0.008 (−0.080, 0.064)	0.830
Academic Year				
1 st Year	ref		ref	
2nd Year	0.108 (−0.094, 0.311)	0.294	0.081 (−0.102, 0.264)	0.383
3rd Year	0.063 (−0.118, 0.245)	0.494	0.008 (−0.166, 0.183)	0.928
4th Year	0.139 (−0.047, 0.325)	0.144	0.024 (−0.171, 0.219)	0.807
5th Year	0.069 (−0.103, 0.242)	0.432	0.022 (−0.185, 0.230)	0.832
Interns	0.216 (0.042, 0.390)	**0.015**	0.114 (−0.125, 0.353)	0.349
University Type				
Public	ref		ref	
Private	0.113 (−0.031, 0.257)	0.122	0.087 (−0.049, 0.223)	0.210
Al-Azhar	−0.064 (−0.167, 0.039)	0.222	−0.022 (−0.128, 0.084)	0.686
Region of University				
Frontier Governorates	ref		ref	
Lower Egypt	0.064 (−0.161, 0.288)	0.579	0.044 (−0.158, 0.246)	0.672
Urban	0.017 (−0.206, 0.241)	0.878	−0.040 (−0.248, 0.167)	0.702
Upper Egypt	−0.005 (−0.248, 0.237)	0.965	0.023 (−0.195, 0.242)	0.835
Nationality				
Egyptian	ref		ref	
Non-Egyptian	0.012 (−0.111, 0.135)	0.849	0.027 (−0.088, 0.142)	0.641
Residence				
Urban	ref		ref	
Rural	−0.015 (−0.096, 0.067)	0.723	−0.016 (−0.094, 0.063)	0.692
English Fluency				
Basic/Conversational	ref		ref	
Intermediate/Working	0.111 (0.004, 0.218)	**0.042**	0.025 (−0.073, 0.123)	0.618
Advanced/Fluent	0.205 (0.092, 0.318)	** <.001**	0.118 (0.008, 0.228)	**0.036**
Cultural Sensitivity Training				
No	ref		ref	
Yes	0.158 (0.079, 0.237)	** <.001**	0.086 (0.007, 0.165)	**0.033**
Extra-curricular Activities Involvement				
No	ref		ref	
Yes	0.126 (0.050, 0.202)	**0.001**	0.030 (−0.043, 0.103)	0.426
Previous Participation in Medical Training Abroad				
No	ref		ref	
Yes	0.146 (0.055, 0.238)	**0.002**	0.017 (−0.076, 0.110)	0.715
Perceived Adequacy of Early Clinical Experience				
Less than Adequate	ref		ref	
Adequate	0.131 (0.045, 0.217)	**0.003**	0.002 (−0.083, 0.087)	0.957
More than Adequate	0.232 (0.094, 0.370)	**0.001**	0.035 (−0.106, 0.175)	0.630
Did not have ECE in my pre-clinical years	−0.069 (−0.209, 0.071)	0.333	−0.045 (−0.172, 0.081)	0.482
Perceived Adequacy of Clinical Training				
Less than Adequate	ref		ref	
Adequate	0.125 (0.034, 0.216)	**0.007**	0.043 (−0.047, 0.133)	0.347
More than Adequate	0.300 (0.169, 0.432)	** <.001**	0.119 (−0.015, 0.253)	0.083
Not in my clinical years yet	−0.097 (−0.227, 0.033)	0.142	−0.155 (−0.287, −0.024)	**0.020**
General educational and research experience	0.256 (0.226, 0.287)	** <.001**	0.120 (0.083, 0.156)	** <.001**
Behaviors/comfort with interactions	−0.068 (−0.100, −0.037)	** <.001**	−0.067 (−0.097, −0.037)	** <.001**
Cognitive awareness	0.250 (0.216, 0.284)	** <.001**	0.080 (0.040, 0.121)	** <.001**
Clinical issues	0.299 (0.267, 0.330)	** <.001**	0.189 (0.148, 0.230)	** <.001**

B = unstandardized regression coefficient;

*CI *confidence interval

Univariate models were run separately for each predictor. The multivariable model included all variables simultaneously. Categorical variables were entered as dummy variables with reference categories as indicated by (ref) in the table

Variance inflation factors (VIFs) for all variables in the multivariable model: Gender 1.09, Region 1.08, University type 1.14, Nationality 1.08, Site of Residence 1.11, English fluency 1.07, Cultural training 1.15, Extracurricular activities 1.10, Training abroad 1.17, ECE adequacy 1.11, Clinical training adequacy 1.15, General Educational/Research Experience 1.27, Behaviors/Comfort with Interactions 1.09, Cognitive Awareness 1.28, Clinical Issues 1.37, Age 1.98, Year of Study 1.20

R^2^ = 0.257; *p*-values < 0.05 are considered statistically significant and are flagged as bold

## Discussion

The ability to deliver effective, patient-centred care to individuals from diverse cultural, religious, linguistic, and social backgrounds is increasingly regarded as a core competency of contemporary medical practice, linked to improved patient satisfaction, treatment adherence, and the reduction of health disparities [30, 31]. Evidence on how well medical students acquire this competency, however, has been generated almost exclusively in high-income Western settings. The present study aimed to address this gap by concurrently examining both cultural awareness and clinical self-perceived cross-cultural skillfulness in a large, multi-institutional sample of medical students in Egypt.

On the skillfulness scale, our sample's mean total self-perceived skillfulness was 3.55, just above the scale midpoint but below the 4-to-5 range regarded as being skilled [5]. Across the skillfulness items, students rated themselves most capable in routine, communication-oriented tasks, such as taking a social history or establishing how a patient wishes to be addressed, and least capable in tasks demanding deeper engagement with cultural and structural complexity, including counselling on CAM, and recognising patient mistrust of the health system. In other words, students appear comfortable with the more straightforward aspects of culturally aware communication while remaining less equipped for its more demanding, context-dependent dimensions [32]. Because our sample was weighted toward senior students – fifth-year students and interns together accounted for more than half of participants – these findings are best read as reflecting cross-cultural preparedness at an advanced, near-graduation stage of training; that even this comparatively experienced cohort reached only intermediate proficiency.

These patterns align closely with the international literature generated using the same instrument. Even at well-resourced North American settings, the gap persists to graduation: at both Harvard, from which the skillfulness sub-scale derives [5], and the University of Michigan [10], fewer than half of students felt adequately skilled on several items by their final year, with the shortfall attributed to gaps in the cross-cultural curriculum and learning climate. The specific weak points recur across very different settings, too as final-year students in Ireland felt confident communicating but least skilled in interpreter use and identifying religious or cultural beliefs; the very items scoring lowest in our sample, with only 20.8% formally trained [11]. Similarly, a Pakistani study echoed the same persistent weakness in alternative-medicine counselling and reported an almost identical skillfulness mean among their senior students using a modified version of the survey [33]. That this shortfall in core cross-cultural skills, and the particular items in which it presents, appears across the United States, Europe, South Asia and Egypt suggests it is not idiosyncratic to any one curriculum but a recurrent, possibly universal, blind spot in undergraduate medical education.

Among the four cultural awareness domains, General Educational and Research Experience received the lowest scores, which may suggest that students perceive their formal educational and research exposure to cultural issues as less developed than other aspects of their cultural awareness. A study from eastern Nepal using a different attitudinal measure offers a useful parallel: although 77.2% of students held good-to-excellent attitudes toward cultural diversity, broadly consistent with the awareness levels seen here, the authors also documented a reliance on superficial knowledge of customs rather than systematic cross-cultural reasoning, alongside a corresponding absence of formal cultural-competence training [12]. This gap is consistent with the historically biomedical orientation of medical education in Egypt, which has traditionally prioritised the basic and clinical sciences over the humanistic and social dimensions of care, of which CCC is one [34]. Together, these observations suggest that the cultural awareness our students report might be acquired through incidental exposure rather than dedicated teaching, consistent with broader patterns in undergraduate cross-cultural education [5, 35].

We found that clinical exposure was related to self-perceived skillfulness, though in a way that points to the quality rather than the mere quantity of experience. Although students in their internship years scored higher in unadjusted analyses, academic year did not remain independently associated with skillfulness after adjustment. Perceived training adequacy showed a similar pattern, with one exception: students who had not yet begun clinical training reported lower skillfulness even after adjustment, though this may simply reflect their inexperience relative to peers already engaged in clinical rotations. This is consistent with Lu et al., who found that cross-cultural preparedness and skillfulness did not improve as students advanced from the preclinical to the clinical stage of training [36], and with a recent Saudi Arabian study reporting no association between year of study and cultural competence among nursing students [37]. This suggests that academic progression alone does not build cross-cultural skill; what matters more, as Seeleman et al. similarly argue, is the comprehensiveness of training itself rather than advancement through it [38]. Separately, students who judged their clinical training less than adequate also scored significantly lower on overall cultural awareness (mCAS) than those rating it more than adequate, raising the possibility that higher-quality clinical training fosters awareness, which may in turn support skillfulness. This is consistent with evidence that immersion in diverse clinical placements builds cultural awareness [39]. Together, these findings support integrating simulated cross-cultural encounters and standardized-patient exercises early in training, rather than deferring cross-cultural learning to clinical rotations alone, where opportunities for structured reflection may be limited [40, 41].

In our study, formal cultural sensitivity training was consistently associated with higher self-perceived skillfulness, retaining significance in the multivariable model despite having been received by only about a third of participants. That an association of this strength persisted after adjustment argues for embedding CCC training within the Egyptian undergraduate curriculum rather than relying on incidental exposure, which our data and the broader literature suggest does not reliably cultivate these skills on its own [38]. We note, however, that students who already hold more favorable attitudes toward cultural competence may be more likely to self-select into such training, so this association should be treated as hypothesis-generating rather than as direct evidence of a training effect. Extracurricular involvement was associated with higher skillfulness in unadjusted analysis but did not retain significance after adjustment, though it was also associated with higher Clinical Issues domain scores, consistent with evidence that volunteering, community work and student organisations expose learners to diverse populations and strengthen teamwork and communication outside the formal curriculum [42]. Prior participation in medical training abroad, although associated with greater skillfulness, was also associated with lower Behaviors/Comfort with Interactions scores. Rather than indicating diminished clinical awareness, this apparent finding may indicate the self-reflective recognition of one's own limitations and discomfort that often accompanies genuine cross-cultural exposure; a hallmark of cultural humility, which prizes ongoing self-critique over a sense of mastery [7, 43]. Taken together, these findings argue for a multimodal educational strategy in which formal curricular training, extracurricular and community engagement, and international or intercultural exposure are treated as complementary components of a single developmental portfolio rather than as isolated initiatives [44].

Among the further factors examined, English fluency was also independently associated with higher self-perceived skillfulness in the multivariable model; mirroring Abdulla et al. who identified English proficiency and plurilingualism as significant correlates of intercultural sensitivity [17]. This fits the role of language as a conduit to cross-cultural communication through two distinct pathways. One interpretation is that greater fluency broadens access to global educational material which are predominantly published in English; it also eases direct communication with the international patients, refugees and tourists encountered in Egyptian clinical settings [16, 45]. However, English fluency may also act as a proxy for unmeasured factors such as secondary schooling type or socioeconomic position, and given that most patients in this setting are Arabic-speaking, we do not interpret this finding as support for English-language instruction as a direct intervention target. Sex was not associated with overall skillfulness, consistent with findings from an Irish medical student cohort [11]. However, female students reported modestly higher Behaviors/Comfort scores (*p* = 0.035). This finding of a modest female advantage confined to interpersonal comfort, rather than to applied skill, aligns with consistent evidence that female medical students report higher empathy and more patient-centred attitudes [46] and also aligns with the significantly greater "respect for cultural differences" held by females in another Egyptian cohort [17]. University type showed a marked difference in Behaviors/Comfort domain, with Al-Azhar students scoring lower than their public-university counterparts; a pattern that may reflect the more homogeneous sociocultural environment and religious framing of medical education at that institution. The absence of significant regional variation in self-perceived skillfulness suggests that the gaps identified here are systemic across the Egyptian medical education system rather than concentrated in particular geographic settings, consistent with the standardised structure of medical training nationally [18].

All four mCAS domains were independently associated with self-perceived skillfulness in the multivariable model, confirming that cultural awareness is meaningfully linked to self-assessed clinical capability, in line with conceptual frameworks that position awareness as one of several interdependent components of cultural competence rather than a standalone endpoint [30–32]. The Clinical Issues domain showed the strongest positive association, whereas Behaviors/Comfort with Interactions was, paradoxically, negatively associated. This negative relationship requires careful interpretation. One plausible but speculative explanation is that students who feel more socially at ease in cross-cultural encounters perceive less need to develop formal clinical skills, or that behavioural comfort reflects a degree of overconfidence that does not correspond to objective competence. This would be consistent with the cultural-humility literature's caution that comfort can mask underlying skill deficits [6, 7], though it remains a hypothesis rather than a finding directly tested by our data. Importantly, this is unlikely to be an anomaly of our data: the original mCAS validation similarly reported low intercorrelations among subscales and a negative correlation involving the Behaviors/Comfort domain, suggesting that this factor captures a distinct psychological construct, namely social ease, rather than clinical knowledge [4]. The correlation matrix supports this reading, with moderate-to-strong positive correlations among General Educational Experience, Cognitive Awareness and Clinical Issues but weak or negative correlations with Behaviors/Comfort. This consistent pattern suggests the mCAS may benefit from further validation in other cultural and linguistic contexts.

The moderate positive correlation between total mCAS and self-perceived skillfulness is consistent with the recognised link between awareness and skill, while its modest magnitude, together with a multivariable model that accounted for roughly a quarter of the variance in skillfulness, suggests that awareness is necessary but not sufficient. Recognising cultural issues is not the same as being equipped to address them at the bedside – a gap also evident among final-year students in Ireland, who strongly endorsed the importance of CCC yet felt unskilled in its more demanding tasks [11]. Cultural skillfulness training and high-quality cross-cultural clinical experience appear to be the bridges through which recognition is translated into competence [32, 47]. Since the awareness domains were themselves the strongest factors independently associated with skillfulness, curricula should target awareness, particularly in the General Educational and Research Experience domain where scores were lowest, alongside applied skill rather than treating either as a substitute for the other.

### Limitations

While this study benefits from a large sample spanning multiple universities and geographic regions, and from the combined use of validated instruments to assess two dimensions of CCC, several limitations should be considered when interpreting its findings. The cross-sectional design precludes causal inference, so the associations reported here, including those between training, awareness and skillfulness, cannot establish directionality and should be interpreted cautiously. Convenience sampling was used here which carries a risk of selection bias: students who chose to complete an unincentivized survey on CCC may have been more engaged with or interested in the topic than non-respondents, which would likely inflate the awareness and self-perceived skillfulness scores reported here relative to the true population average. All outcomes were self-reported and therefore subject to social-desirability and self-assessment bias, which is a recognised concern in cross-cultural competence research, where perceived and demonstrated ability may diverge. The sample was weighted toward senior students, which limits generalisability to earlier stages of training and may attenuate observed year-on-year trends. In addition, the number of students from private universities was relatively small, which may limit the representativeness and precision of estimates for this sector. Also, socioeconomic indicators (e.g., parental education, secondary schooling type, family income) were not collected, and English fluency, which was independently associated with skillfulness, may partly reflect these unmeasured factors rather than a directly modifiable target.

Finally, the mCAS was developed and validated in Sweden, Hong Kong, and the United States, and the skillfulness sub-scale in the United States medical-student population. Although both instruments have demonstrated transportability to new settings, their application in an Arabic-speaking context is itself novel, constituting both a limitation and a contribution. The three items modified for cultural and linguistic appropriateness were reviewed informally for content suitability and evaluated using corrected item-total correlations rather than a full psychometric re-validation. While these checks did not suggest that the modifications compromised item performance, formal re-validation of the adapted items in this population would strengthen future work.

## Conclusions

In this large, multi-university sample of Egyptian medical students, self-perceived cultural skillfulness was at an intermediate level, with the lowest self-ratings for counselling patients on complementary and alternative medicine and recognising patient mistrust, followed by identifying cultural customs affecting care. Cultural awareness showed a modest positive correlation with self-perceived skillfulness. In adjusted analyses, previous cultural sensitivity training, advanced English fluency, and the cultural awareness domains were independently associated with skillfulness, though together these factors explained only around a quarter of its variance, suggesting that awareness and applied skill are related but distinct, and that awareness alone does not ensure skillful cross-cultural practice. Given the cross-sectional design and reliance on self-report, these associations should be interpreted as correlational rather than causal. Cultural sensitivity training and the targeted strengthening of cultural awareness are potential curricular priorities warranting prospective evaluation, rather than treating incidental clinical exposure as sufficient on its own. Longitudinal or interventional studies are needed to determine whether such training translates into observed clinical competence.

## Supplementary Information

Supplementary Material 1.

## Data Availability

The data of this study is available from the corresponding author upon reasonable request.

## Abbreviations

ANOVA: Analysis of Variance
CAM: Complementary and Alternative Medicine
CCC: Cross-Cultural Care
CCCS: Cross-Cultural Care Survey
CI: Confidence Interval
ECCC Consortium: Egyptian Cross-Cultural Care Consortium
IRB: Institutional Review Board
mCAS: Modified Cultural Awareness Scale
Q-Q: Quantile-Quantile
SD: Standard Deviation
STROBE: Strengthening the Reporting of Observational Studies in Epidemiology
VIFs: Variance Inflation Coefficients
