# Supplementary material for: Cross-cultural care awareness and self-perceived skillfulness among medical students in Egypt: a national study
Source: BMC Med Educ. 2026 Oct 31;26:1760. doi: 10.1186/s12909-026-10494-2 (PMC13723094; doi:10.1186/s12909-026-10494-2)
Supplement: Supplementary file 1 [file 12909_2026_10494_MOESM1_ESM.docx]

STROBE Statement—Checklist of items that should be included in reports of ***cross-sectional studies***

“Cross-cultural care skillfulness and cultural awareness among medical students in Egypt: a multicenter cross-sectional study”

|  | **Item No** | **Recommendation** | **Section in the manuscript** |
| --- | --- | --- | --- |
| **Title and abstract** | 1 | (*a*) Indicate the study’s design with a commonly used term in the title or the abstract | Title of manuscript; Abstract — Methods paragraph, P1 |
|  |  | (*b*) Provide in the abstract an informative and balanced summary of what was done and what was found | Abstract (Background, Methods, Results, Conclusion), P1 |
| **Introduction** | | |  |
| Background/rationale | 2 | Explain the scientific background and rationale for the investigation being reported | Introduction — paragraphs 1–4 |
| Objectives | 3 | State specific objectives, including any prespecified hypotheses | Introduction — final paragraph ('this study assessed…') |
| **Methods** | | |  |
| Study design | 4 | Present key elements of study design early in the paper | Methods — 'Study design' subsection  (Multicentric, descriptive cross-sectional study with an analytical component) |
| Setting | 5 | Describe the setting, locations, and relevant dates, including periods of recruitment, exposure, follow-up, and data collection | Methods — 'Study design, population, and setting' subsections  (Conducted between November 2025 and March 2026; across 14 Egyptian universities spanning 3 higher education sectors: public, private, and Al-Azhar) |
| Participants | 6 | (*a*) Give the eligibility criteria, and the sources and methods of selection of participants | Methods — 'Setting and population' subsections  (inclusion/exclusion criteria listed explicitly) |
| Variables | 7 | Clearly define all outcomes, exposures, predictors, potential confounders, and effect modifiers. Give diagnostic criteria, if applicable | Methods — 'Study tools' (Skillfulness via CCCS , Cultural awareness via mCAS with domains defined; sociodemographic predictors listed in questionnaire) |
| Data sources/ measurement | 8* | For each variable of interest, give sources of data and details of methods of assessment (measurement). Describe comparability of assessment methods if there is more than one group | Methods — 'Study tools' subsection  (Self-administered online questionnaire, scales described; scoring defined; Cronbach's α = 0.89 reported in our analysis, Prior pilot testing conducted ) |
| Bias | 9 | Describe any efforts to address potential sources of bias | Methods — 'Sampling and data collection approach' subsection  (Stratified convenience sampling utilized to ensure broad national representation across 4 main regions; design effect of 2.5 applied to sample size calculation; Google forms restricted to one response per account to prevent duplicates) |
| Study size | 10 | Explain how the study size was arrived at | Methods — 'Sample size' subsection  (R samplingbook package; pilot SD=0.64; margin of error = 0.05; design effect=2.5; minimum required n=1445; final n=1645) |
| Quantitative variables | 11 | Explain how quantitative variables were handled in the analyses. If applicable, describe which groupings were chosen and why | Methods — 'Statistical analysis' subsection  (Continuous variables summarized as means/SDs after checking normality; Likert items summarized continuously and categorically). |
| Statistical methods | 12 | (*a*) Describe all statistical methods, including those used to control for confounding | Methods — 'Statistical analysis' subsection ((Pearson correlation coefficients, independent samples t-tests; one-way ANOVA, two-stage linear regression modeling) |
|  |  | (*b*) Describe any methods used to examine subgroups and interactions | Methods — 'Statistical analysis' subsection  (Comparisons of mean scores across sociodemographic characteristics using t-tests for two groups and ANOVA for multi-group comparisons) |
|  |  | (*c*) Explain how missing data were addressed | Methods — 'Setting' Subsection  (57 entries were explicitly excluded from the preliminary dataset for providing incomplete responses) |
|  |  | (*d*) If applicable, describe analytical methods taking account of sampling strategy | Methods — 'Sampling and data collection approach' and 'Statistical analysis' subsections  (Total sample distributed proportionately based on official student census; categorical variables entered as dummy variables in regression) |
|  |  | (*e*) Describe any sensitivity analyses | Not done |
| **Results** | | |  |
| Participants | 13* | (a) Report numbers of individuals at each stage of study—eg numbers potentially eligible, examined for eligibility, confirmed eligible, included in the study, completing follow-up, and analysed | Results — 'Participants' Characteristics' (1,702 completed questionnaire; 57 excluded [missing data or non selected universities]; 1645in final analysis) |
|  |  | (b) Give reasons for non-participation at each stage | Not applicable |
|  |  | (c) Consider use of a flow diagram | Not included (not applicable in sampling design chosen) |
| Descriptive data | 14* | (a) Give characteristics of study participants (eg demographic, clinical, social) and information on exposures and potential confounders | Results — Participants' Characteristics and Table 1 (full sociodemographic and clinical training perceptions) |
|  |  | (b) Indicate number of participants with missing data for each variable of interest | Not reported |
| Outcome data | 15* | Report numbers of outcome events or summary measures | Results — Cross-Cultural Care Skillfulness & Table 2 (Reports mean overall skillfulness score of 3.55 ± 0.78, item-level score ranges [3.42 to 3.70], and percentages of participants receiving a "Skilled" rating). Sociodemographic Variations (Reports mean mCAS of 4.69 ± 0.75) |
| Main results | 16 | (*a*) Give unadjusted estimates and, if applicable, confounder-adjusted estimates and their precision (eg, 95% confidence interval). Make clear which confounders were adjusted for and why they were included | Results — Predictors of Cross-Cultural Care Skillfulness & Table 5 (Reports multivariable linear regression adjusting for sociodemographic factors; provides B coefficients and p-values for independent predictors like advanced English fluency [B = 0.118], cultural sensitivity training [B = 0.086], and clinical-year status [B = -0.155]) |
|  |  | (*b*) Report category boundaries when continuous variables were categorized | Results — Cross-Cultural Care Skillfulness (Reports the categorical grouping of "Skilled" participants) |
|  |  | (*c*) If relevant, consider translating estimates of relative risk into absolute risk for a meaningful time period | Not applicable — cross-sectional study; no relative risk estimates reported |
| Other analyses | 17 | Report other analyses done—eg analyses of subgroups and interactions, and sensitivity analyses | Results — Correlations Between mCAS Domains, Skillfulness, and Age and Table 3 (Pearson correlations). Results – Sociodemographic Variations in Cultural Awareness and Skillfulness and Table 4 (Subgroup analyses comparing total scores across sex, academic year, English proficiency, and experiential factors) |
| **Discussion** | | |  |
| Key results | 18 | Summarise key results with reference to study objectives | Discussion — Paragraphs 1, 2, & 4 (Summarizes the objective to examine awareness and skillfulness; reports the mean skillfulness of 3.55, noting higher capability in routine tasks vs. lower capability in complex tasks like CAM counseling. General Educational and Research Experience scored lowest among domains) |
| Limitations | 19 | Discuss limitations of the study, taking into account sources of potential bias or imprecision. Discuss both direction and magnitude of any potential bias | Discussion — Limitations subsection  (cross-sectional design; self-report and social-desirability bias, online sampling bias, convenience sampling, senior-weighted sample, small private university representation, and the novelty/limitation of using instruments validated in Western/Asian settings in an Arabic-speaking context ) |
| Interpretation | 20 | Give a cautious overall interpretation of results considering objectives, limitations, multiplicity of analyses, results from similar studies, and other relevant evidence | Discussion — body paragraphs: 3, 5, 6, 8, and 9 (Interprets findings in the context of international literature; concludes that academic progression alone does not build skill, but quality of training does; interprets the negative association of the Behaviors/Comfort domain as potential overconfidence masking deficits; concludes that awareness is necessary but not sufficient for clinical competence) |
| Generalisability | 21 | Discuss the generalisability (external validity) of the study results | Discussion — Paragraph 7 and Limitations subsection (Paragraph 10) |
| **Other information** | | |  |
| Funding | 22 | Give the source of funding and the role of the funders for the present study and, if applicable, for the original study on which the present article is based | Funding — No funding or grants were received for this research |

*Give information separately for exposed and unexposed groups.

**Note:** An Explanation and Elaboration article discusses each checklist item and gives methodological background and published examples of transparent reporting. The STROBE checklist is best used in conjunction with this article (freely available on the Web sites of PLoS Medicine at http://www.plosmedicine.org/, Annals of Internal Medicine at http://www.annals.org/, and Epidemiology at http://www.epidem.com/). Information on the STROBE Initiative is available at www.strobe-statement.org.
